# Development of a fluorogenic small substrate for dipeptidyl peptidase-4

**DOI:** 10.3762/bjoc.13.267

**Published:** 2017-12-14

**Authors:** Futa Ogawa, Masanori Takeda, Kanae Miyanaga, Keita Tani, Ryuji Yamazawa, Kiyoshi Ito, Atsushi Tarui, Kazuyuki Sato, Masaaki Omote

**Affiliations:** 1 Faculty of Pharmaceutical Sciences, Setsunan University, 45-1 Nagaotoge-cho, Hirakata, Osaka 573-0101, Japan; 2 Division of Natural Sciences, Osaka Kyoiku University, Asahigaoka, Kashiwara, Osaka 582-8582, Japan

**Keywords:** dipeptidyl peptidase-4, fluorogenic substrate, fluorometry, small fluorescent molecule

## Abstract

A series of aniline and *m*-phenylenediamine derivatives with electron-withdrawing 3,3,3-trifluoropropenyl substituents were synthesized as small and chemically stable fluorescent organic compounds. Their fluorescence performances were evaluated by converting 2,4-disubstituted aniline **1** to the non-fluorescent dipeptide analogue H-Gly-Pro-**1** for the use as a fluorogenic substrate for dipeptidyl peptidase-4 (DPP-4). The progress of the enzymatic hydrolysis of H-Gly-Pro-**1** with DPP-4 was monitored by fluorometric determination of **1** released into the reaction medium. The results suggest that **1** could be used as fluorophore in OFF–ON-type fluorogenic probes.

## Introduction

Fluorescent organic probes have become indispensable for bio-imaging or molecular imaging to visualize the presence of species such as particular enzymes and biologically important inorganic ions. In biomedicine, these probes are used for the detection of a wide range of significant biomarkers and are essential for the diagnosis of critical diseases [1–3]. Selective and accurate determinations of specific enzymes, including β-galactosidase [4–6], exoglycosidases [7], cyclooxygenases [8], and others [9–13], have been achieved using the OFF–ON-type fluorogenic probes with fluorescence that can be turned on by enzymatic transformations. These fluorescent probes can act as good sensors for enzymes but the fluorophore unit is usually large because fused aromatic rings and an elongated π-conjugated system are necessary to impart appropriate fluorescent properties to the probe [14]. The molecular size of the fluorescent probe is therefore large, which is sometimes inconvenient in terms of membrane permeability, water solubility, and inhibition of inherent interactions between the probe molecule and the target enzyme. We have developed a new OFF–ON probe with a small fluorescent core unit. The same approach was recently used to produce a fluorophore for a push–pull system, which has a single benzene ring substituted by both electron-donating and electron-withdrawing groups [15–17]. We focused on the 3,3,3-trifluoropropenyl (TFPE) group, which is an electron-withdrawing substituent that is free of oxygen atoms, which would form hydrogen bonds in protic solvents. We assumed that the use of the oxygen-free TFPE group would eliminate hydrogen bonding with protic solvents, even water, to avoid attenuation of the fluorescent characteristics of the fluorophore. In this paper, we report the synthesis of a new fluorophore with TFPE groups and its application as an OFF–ON-type fluorogenic probe for determining dipeptidyl peptidase-4 (DPP-4) activity.

## Results and Discussion

### Synthesis of fluorescent compounds as fluorophores

It has been reported that the introduction of a TFPE group changes the properties of electron-rich aromatics and leads to a good fluorophore structure. This suggests that substitution with TFPE would be a reasonable approach to producing new fluorescent molecules. However, there are few methods for synthesizing TFPE-substituted compounds. In a previous study, we achieved the facile introduction of TFPE groups via Hiyama cross-coupling reactions of (*E*)-trimethyl-(3,3,3-trifluoroprop-1-enyl)silane and iodoanilines. This straightforward method is useful for TFPE introduction and a broad range of iodoanilines can be used without protection of the amino group. We used this approach to synthesize several aniline derivatives containing TFPE groups. The data in Table 1 show that Hiyama cross-coupling reactions gave the desired TFPE-anilines and TFPE-phenylenediamines (**1**–**4**) in moderate to good yields. This method has the advantage that complete substitution of diiodo- and triiodoarenes can proceed to give disubstituted (**1** and **3**) and trisubstituted (**2** and **4**) products in one synthetic operation.

**Table 1:** Synthesis of TFPE-anilines **1**–**4** via Hiyama cross-coupling reactions of iodoanilines with (*E*)-trimethyl(3,3,3-trifluoroprop-1-enyl)silane.^a^

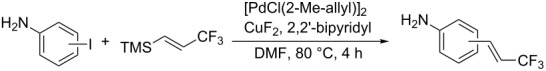			

Entry	ArI	Product	Yield (%)^b^

1	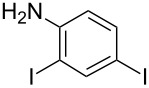	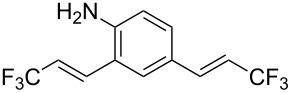 **1**	89
2	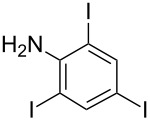	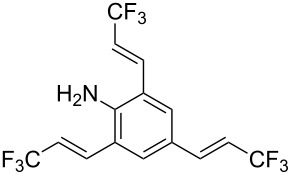 **2**	54
3	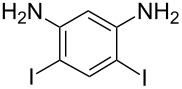	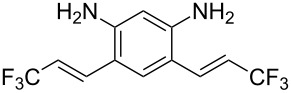 **3**	70
4	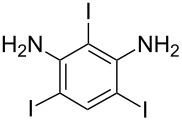	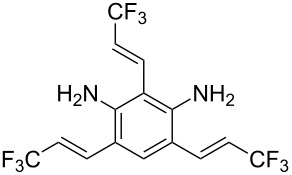 **4**	20

^a^Reaction conditions: ArI (1.0 mmol), (*E*)-trimethyl(3,3,3-trifluoroprop-1-enyl)silane (2 equiv), [PdCl(2-Me-allyl)]_2_ (10 mol %), CuF_2_ (2 equiv), 2,2'-bipyridyl (2 equiv), DMF (6 mL). ^b^Isolated yield.

**Figure 1:**
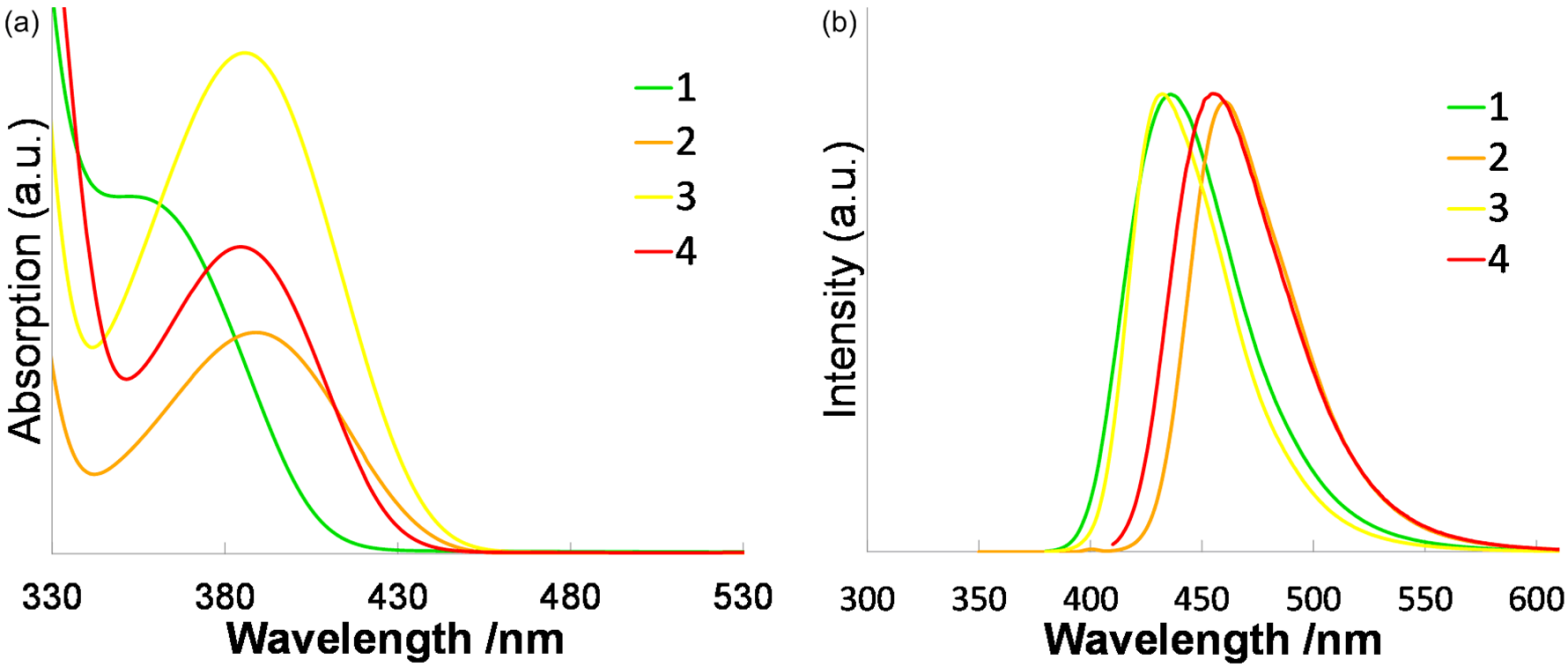
(a) UV–vis absorption and (b) fluorescence spectra of **1**–**4** in THF.

**Figure 2:**
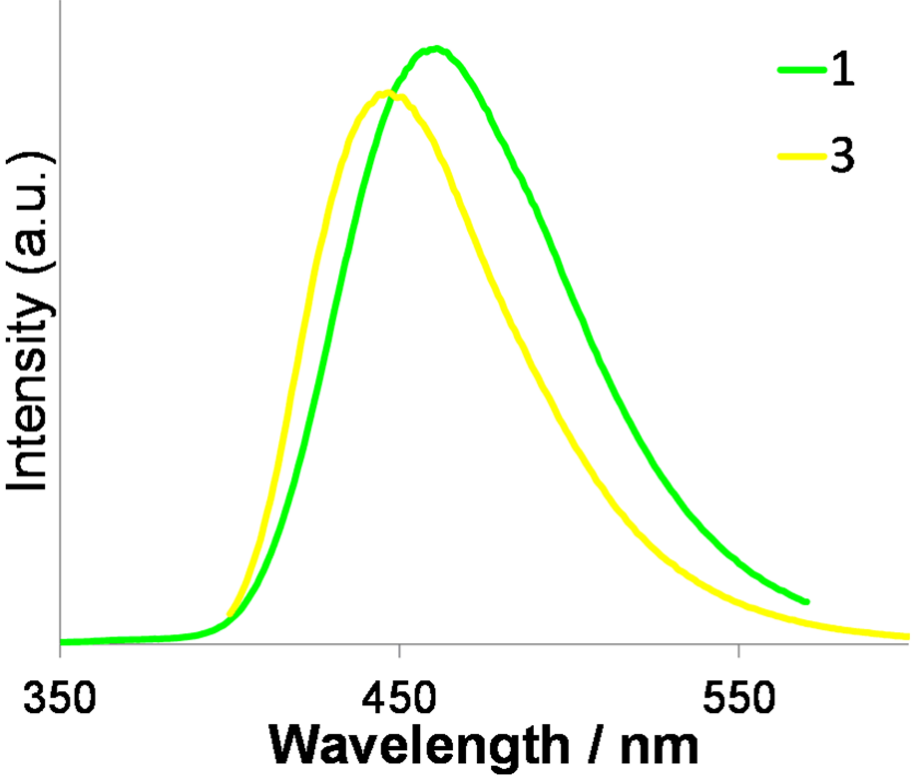
Fluorescence spectra of **1** and **3** in H_2_O:DMSO (9:1).

Next, the fluorescence profiles of these products were investigated. The results are summarized in Figure 1 and Table 2. The number of amino groups and TFPE groups introduced into compounds **1**–**4** significantly affected the fluorescence profiles in THF solution. TFPE-anilines **1** and **2** gave moderate fluorescence quantum yields of 0.31 and 0.30, respectively. In comparison with these, the fluorescence quantum yields of TFPE-phenylenediamine **3** was slightly lower, namely 0.25. The introduction of one more TFPE group into **3**, to give **4**, further decreased the quantum yield to 0.03. In spectroscopic terms, the fluorescence emission peaks of these compounds red-shifted with increasing TFPE substitution. Additionally, a large Stokes shift was observed for **1**, which is important in view of the biomedical use of fluorescent compounds. Next, we changed the solvent from THF to H_2_O/DMSO (9:1) as shown in Figure 2 and Table 3. The fluorescence quantum yield of a fluorophore generally decreases significantly in an aqueous solvent because of release of energy in the excited state of the fluorophore by forming hydrogen bonds with water. However, in the cases of **1** and **3**, the decreases in the fluorescence quantum yield were small, namely 0.27 and 0.20, respectively. This is reasonable for **1** and **3** because the oxygen-free TFPE group is less likely to form hydrogen bonds, even in aqueous solution. Additionally, the emission peak of **1** is red-shifted from 436 to 461 nm. On the basis of the properties of **1**–**4**, we chose **1** for subsequent experiments involving enzymatic reactions.

**Table 2:** UV–vis absorption and fluorescence data for **1**–**4** in THF.^a^

	λ_abs,peak_ (nm)	ε (M^−1^cm^−1^)	λ_fl,peak_ (nm)	φ^b^

**1**	359	4371	436	0.31
**2**	388	5763	460	0.30
**3**	373	7913	433	0.25
**4**	385	7004	455	0.03

^a^Measurement conditions:1.0 × 10^−5^ M in THF, excitation at λ = 370 nm for **1** and **3**, 400 nm for **2** and **4**. ^b^Fluorescence quantum yield.

**Table 3:** Fluorescence data for **1**–**3** in H_2_O:DMSO (9:1).^a^

	λ_fl,peak_ (nm)	φ^b^

**1**	461	0.27
**2**	-^c^	-^c^
**3**	447	0.20

^a^Measurement conditions: 1.0 × 10^−5^ M in H_2_O:DMSO (9:1), excitation at λ = 330 nm for **1** and **3**. ^b^Fluorescence quantum yield. ^c^**2** is insoluble in H_2_O:DMSO (9:1).

### Design of fluorogenic probes

**Scheme 1:**
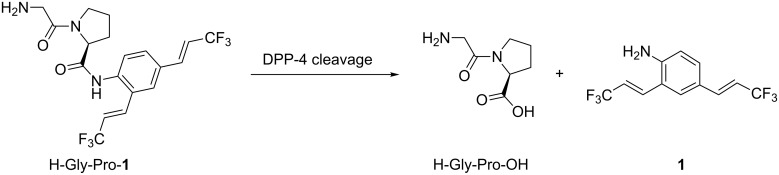
Use of **1** as fluorogenic probe for DPP-4 activity.

Fluorogenic OFF–ON-type probes for enzymes should be designed carefully to ensure that the fluorescence profiles before and after the enzymatic reaction are different, ideally, non-fluorescence and intensive fluorescence, respectively. On the basis of the structural features of **1**, we expected that its fluorescence would disappear as a result of peptide derivatization of the amino group, because electron donation by the amino group would be attenuated and the fluorescence push–pull system would be destroyed. To confirm this, we modified **1** to provide a peptidic substrate for an enzyme. The serine protease DPP-4 was used as the test enzyme because its substrate specificity is clear: it hydrolyses the C-terminal of proline or alanine second to the N-terminal of the peptide [18]. Additionally, DPP-4 is a significant biomarker for the progress of diabetes, and several chromogenic or fluorogenic substrates for DPP-4 have been developed [19–21]. We therefore evaluated **1** as a fluorogenic probe for DPP-4 activity using the peptide analogue H-Gly-Pro-**1** as a substrate for DPP-4. The experimental strategy is summarized in Scheme 1; non-fluorescent H-Gly-Pro-**1** is hydrolysed specifically by DPP-4 to release **1**, enabling fluorometric measurements.

### Synthesis of the fluorogenic substrate for DPP-4

**Scheme 2:**
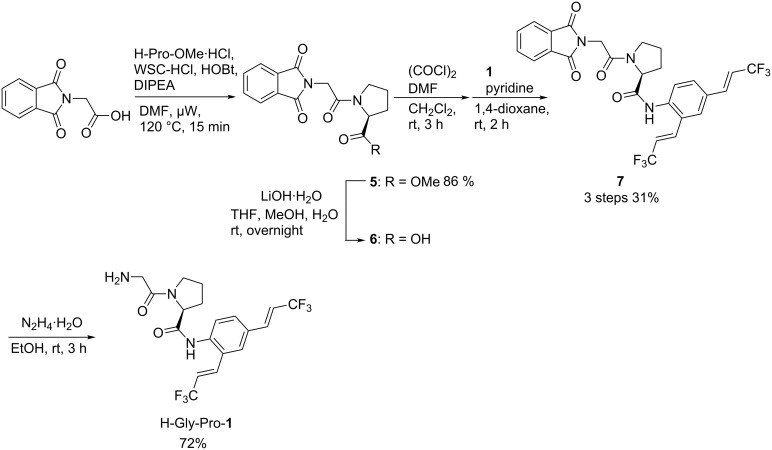
Synthesis of fluorogenic probe H-Gly-Pro-**1**.

The synthesis of H-Gly-Pro-**1** is shown in Scheme 2. In the synthesis, N-protected glycine was condensed with proline methyl ester under microwave irradiation. Subsequent hydrolysis of the ester gave N-protected Gly-Pro-OH (**6**). Condensation of dipeptide **6** with **1** was achieved using the corresponding acid chloride. Deprotection of the amino group was achieved by treatment with hydrazine, without disturbing the peptide bond structure, to give H-Gly-Pro-**1**.

### DPP-4 activity measurement

**Figure 3:**
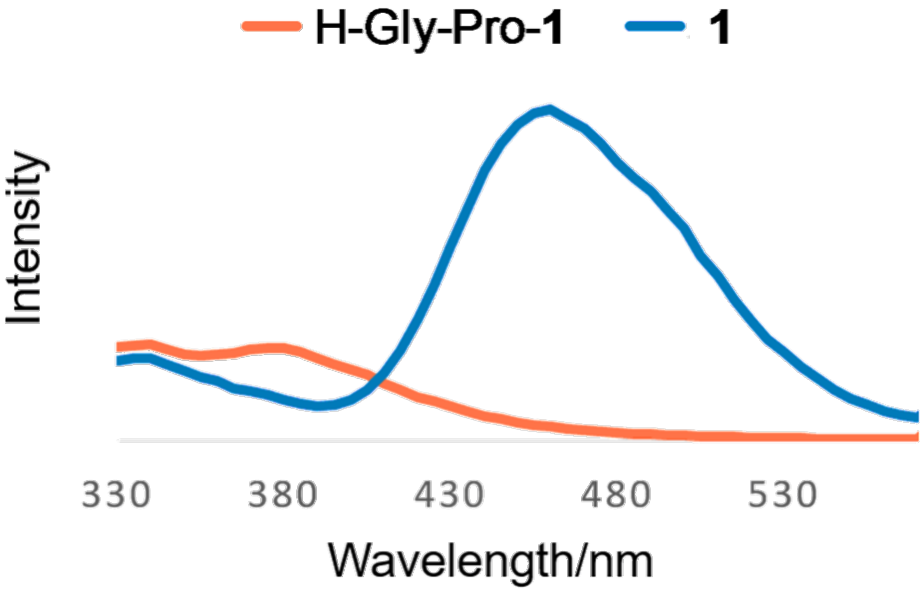
Fluorescence spectra of **1** and H-Gly-Pro-**1**. Measurement conditions: 1.0 × 10^−5^ M in H_2_O:DMSO (9:1), excitation at λ = 300 nm.

The fluorescence spectra of **1** and H-Gly-Pro-**1** were recorded to confirm their fluorescence profiles; the spectra are shown in Figure 3. As expected, H-Gly-Pro-**1** was non-fluorescent because of the modification of the amino group to an amide group. The loss of fluorescence is the result of attenuation of the electron-donating effect of the amino group to the benzene ring. In particular, a large difference between the fluorescence emissions of **1** and H-Gly-Pro-**1** at around 460 nm was observed. H-Gly-Pro-**1** can therefore be used as a fluorogenic substrate for DPP-4 to minimize spectral interference from the background state.

**Figure 4:**
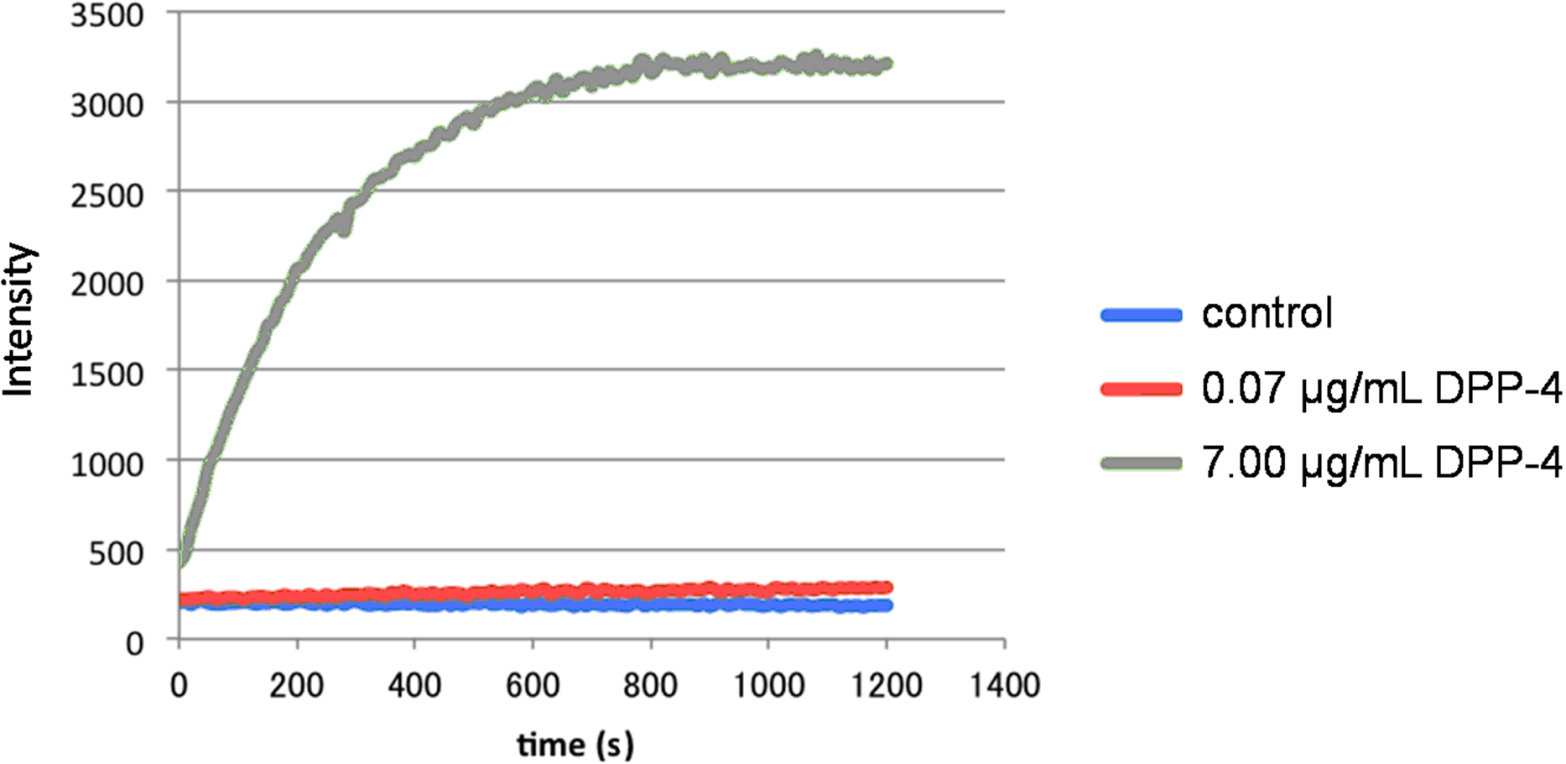
Fluorescence intensity changes of H-Gly-Pro-**1** on addition of DPP-4.

Next, we determined whether or not DPP-4 could hydrolyse H-Gly-Pro-**1**. The hydrolysis experiments were conducted using H-Gly-Pro-**1** with DPP-4 concentrations of 0.07 and 7.00 μg/mL, and a control. The results are shown in Figure 4. Enzymatic hydrolysis occurred when the DPP-4 concentration was 7.00 μg/mL, as shown by the increased fluorescence intensity as the reaction proceeded. In contrast, hydrolysis was not observed in the absence of DPP-4 (control). The reaction did not proceed at a low concentration of DPP-4 (0.07 μg/mL). These results suggest that H-Gly-Pro-**1** could be used as an OFF–ON-type fluorogenic substrate for enzymatic reactions of DPP-4, with release of **1** as a fluorophore. In comparison with existing fluorogenic substrates containing rhodamine fluorophore, non-fluorescent characteristic of H-Gly-Pro-**1** have negligible background on the fluorometry to permit easy and clear identification of reaction progress. On the other hand, H-Gly-Pro-**1** needs some improvements in terms of the reaction rate for this enzymatic reaction.

## Conclusion

We synthesized the small fluorescent compounds **1**–**4**. Among them, **1** showed a good fluorescence profile despite its small size. The use of **1** as a probe was investigated using the derivative H-Gly-Pro-**1** as a fluorogenic substrate for DPP-4. The enzymatic activity of DPP-4 was confirmed by measuring the fluorescence intensity of the reaction medium, in which fluorescent **1** accumulated as the reaction progressed. H-Gly-Pro-**1** behaves as an OFF–ON-type fluorogenic substrate for DPP-4. Further studies of the synthesis of different TFPE-aniline derivatives and fluorogenic substrates for other enzymes are underway.

## Experimental

### General information

All experiments were carried out under an argon atmosphere in flame-dried glassware using standard inert techniques for introducing reagents and solvents, unless otherwise noted. *N,N*-Dimethylformamide (DMF) was distilled over calcium hydride and stored in a bottle with activated molecular sieves (4 Å). All commercially available materials were used as received without further purification. ^1^H NMR, ^13^C NMR and ^19^F NMR spectra were measured on a JEOL ECZS 400S spectrometer (^1^H: 400MHz, ^13^C: 100 MHz, ^19^F: 376 MHz). Chemical shifts of ^1^H NMR and ^13^C NMR are reported in parts per million from tetramethylsilane (TMS), used as an internal standard at 0 ppm. Chemical shifts of ^19^F NMR are reported in parts per million from trichlorofluoromethane (CFCl_3_), used as an internal standard at 0 ppm. All dates are reported as follows: chemical shifts, relative integration value, multiplicity (s = singlet, d = doublet, t = triplet, q = quartet, m = multiplet), and coupling constants (Hz). High-resolution mass spectroscopy (HRMS) experiments were performed with a double-focusing mass spectrometer with EI. Melting points were measured on Yanaco melting point apparatus MP-500V without correction. Microwave reactions were performed in microwave tubes with clip lids using a Biotage Initiator microwave reactor.

### Typical procedure for the Hiyama cross-coupling reaction

Analogous as described in [24]. In a glovebox purged with argon gas, iodo aniline (1.0 mmol), (2-methylallyl)palladium(II) (0.1 mmol), CuF_2_ (2.0 mmol), and 2,2’-bipyridyl (2.0 mmol) were placed in a flask. To the flask were added anhydrous DMF (6.0 mL) and (*E*)-trimethyl(3,3,3-trifluoroprop-1-enyl)silane (2.0 mmol), and mixture was stirred at 80 °C. After the reaction mixture was stirred for 4 h, it was poured into ice water. The mixture was extracted with CH_2_Cl_2_, and the organic layer was dried over anhydrous MgSO_4_. After the solid was filtered, the solvent was removed in vacuo, and the residue was purified by silica gel column chromatography to give product.

**2,4-Bis[(*E*)-3,3,3-trifluoroprop-1-enyl]aniline (1)**: The title product was purified by column chromatography and was obtained in 89% yield (251 mg). A light yellow solid: mp 106–107 °C (recrystallized from AcOEt and hexane); ^1^H NMR (CDCl_3_) δ 4.05 (s, 2H), 6.04 (qd, *J* = 6.6, 16.2 Hz, 1H), 6.19 (qd, *J* = 6.5, 15.8 Hz, 1H), 6.72 (d, *J* = 8.2 Hz, 1H), 7.03 (qd, *J* = 2.1, 16.2 Hz, 1H), 7.20 (qd, *J* = 2.1, 15.8 Hz, 1H), 7.30 (dd, *J* = 1.8, 8.2 Hz, 1H), 7.36 (d, *J* = 1.8 Hz, 1H); ^13^C NMR (CDCl_3_) δ 112.9 (q, *J* = 33.7 Hz), 116.8, 117.9 (q, *J* = 33.7 Hz), 119.2, 123.3 (q, *J* = 269.4 Hz), 123.9 (q, *J* = 269.4 Hz), 124.4, 127.9, 132.7 (q, *J* = 6.7 Hz), 136.8 (q, *J* = 6.7 Hz), 146.3; ^19^F NMR (CDCl_3_) δ 89.84 (dd, *J* = 2.2, 6.5 Hz, 3F), 90.47 (dd, *J* = 2.2, 6.5 Hz, 3F); MS *m*/*z*: M^+^ 281, 242, 211; HRMS *m*/*z*: M^+^ calcd for C_12_H_9_F_6_N, 281.0639; found, 281.0634.

**2,4,6-Tris((*E*)-3,3,3-trifluoroprop-1-en-1-yl)aniline (2)**: The title product was purified by column chromatography and was obtained in 54% yield (203 mg). A light yellow solid: mp 119–120 °C (recrystallized from AcOEt and hexane); ^1^H NMR (CDCl_3_) δ 4.20 (s, 2H), 6.11 (qd, *J* = 6.5, 16.0 Hz, 1H), 6.20 (qd, *J* = 6.4, 16.0 Hz, 2H), 7.05 (qd, *J* = 2.1, 16.0 Hz, 1H), 7.21 (qd, *J* = 2.1, 16.0 Hz, 2H), 7.40 (s, 2H); ^13^C NMR (CDCl_3_) δ 114.1 (q, *J* = 34.0 Hz), 119.8 (q, *J* = 33.6 Hz), 121.1, 123.0 (q, *J* = 269.4 Hz), 124.3, 124.7 (q, *J* = 269.2 Hz), 128.7, 132.3 (q, *J* = 6.8 Hz), 136.2 (q, *J* = 6.7 Hz), 143.9; ^19^F NMR (CDCl_3_) δ 89.59 (dd, *J* = 2.2, 6.5 Hz, 6F), 90.24 (dd, *J* = 2.2, 6.5 Hz, 3F); MS *m*/*z*: M^+^ 375, 316, 266; HRMS *m*/*z*: M^+^ calcd for C_15_H_10_F_9_N_3_, 375.0670; found, 375.0668.

**4,6-Bis((*E*)-3,3,3-trifluoroprop-1-en-1-yl)-1,3-diaminobenzene (3)**: The title product was purified by column chromatography and was obtained in 70% yield (183.7 mg). A light brown solid: mp 165–167 °C (recrystallized from AcOEt and hexane); ^1^H NMR (CDCl_3_) δ 3.95 (s, 4H), 6.00 (s, 1H), 6.01 (qd, *J* = 6.6, 16.0 Hz, 2H), 7.09 (qd, *J* = 2.1, 16.0 Hz, 2H), 7.27 (s, 1H); ^13^C NMR (CDCl_3_) δ 102.3, 111.4, 113.9 (q, *J* = 33.4 Hz), 123.8 (q, *J* = 268.8 Hz), 128.6, 132.4 (q, *J* = 6.7 Hz), 147.5; ^19^F NMR (CDCl_3_) δ 89.83 (dd, *J* = 2.2, 6.5 Hz, 3F), 90.47 (dd, *J* = 2.2, 6.5 Hz, 3F). MS *m*/*z*: M^+^ 296, 277, 257, 226, 187; HRMS *m*/*z*: M^+^ calcd for C_12_H_10_F_6_N_2_, 296.2116; found, 296.0749.

**2,4,6-Tris((*E*)-3,3,3-trifluoroprop-1-en-1-yl)-1,3-diaminobenzene (4)**: The title product was purified by column chromatography and was obtained in 20% yield (78 mg). A orange solid: mp 196 °C (recrystallized from AcOEt and hexane); ^1^H NMR (CDCl_3_) δ 4.12 (s, 4H), 6.04 (qd, *J* = 6.4, 15.9 Hz, 2H), 6.21 (qd, *J* = 6.1, 16.4 Hz, 1H), 7.02 (qd, *J* = 2.0, 16.4 Hz, 1H), 7.11 (qd, *J* = 2.0, 15.9 Hz, 2H), 7.28 (s, 1H); ^13^C NMR (CDCl_3_) δ 105.7, 111.1, 115.4 (q, *J* = 33.7 Hz), 122.6 (q, *J* = 270.3 Hz), 123.6 (q, *J* = 269.0 Hz), 124.9 (q, *J* = 33.7 Hz), 128.6, 131.0 (q, *J* = 6.7 Hz), 132.3 (q, *J* = 6.7 Hz), 144.5; ^19^F NMR (CDCl_3_) δ 89.18 (dd, *J* = 1.5, 5.8 Hz, 3F), 90.40 (dd, *J* = 1.5, 6.5 Hz, 6F); MS *m*/*z*: M^+^ 390, 321; HRMS *m*/*z*: M^+^ calcd for C_15_H_11_F_9_N_3_, 390.0779; found, 390.0783.

### Fluorogenic substrate synthesis

**1-[2-(1,3-Dihydro-1,3-dioxo-2*H*-isoindol-2-yl)acetyl]-L-proline methyl ester (5): ***N-*Phtaloylglycine (4 mmol), 1-hydroxybenzotriazole (4.4 mmol) and *N*-ethyl-*N*’(3-dimethylaminopropyl)carbodiimide hydrochloride (10 mmol) were placed in a microwave vial. To the microwave vial was added anhydrous DMF (8 mL) and anhydrous *N*,*N*-diisopropylethylamine (20 mmol), and the mixture was stirred at room temperature. After the reaction mixture was stirred for 5 min, to the microwave vial was added L-proline methyl ester hydrochloride (4.8 mmol) and the mixture was heated by microwave irradiation for 20 min at 120 °C. The resulting mixture was quenched with water and extracted with AcOEt. The AcOEt layer was washed with brine and dried over MgSO_4_. The solvent was removed in vacuo and the residue was purified by column chromatography to give 1-[2-(1,3-dihydro-1,3-dioxo-2*H*-isoindol-2-yl)acetyl]-L-proline methyl ester (**5**) in 86% yield (1.09 g, 3.4 mmol). A white solid: mp 166–167 °C ; ^1^H NMR (CDCl_3_) δ 1.90–2.36 (m, 4H), 3.60–3.83 (m, 5H), 4.40 (d, *J* = 16.5 Hz, 1H), 4.51–4.61 (m, 2H), 7.70–7.73 (m, 2H), 7.85–7.88 (m, 2H); ^13^C NMR (CDCl_3_) δ 24.8, 28.9, 39.7, 46.2, 52.3, 59.1, 123.5, 132.2, 134.0, 164.4, 167.8, 172.1; MS *m*/*z*: M^+^ 316, 284, 257, 188, 160, 128; HRMS *m*/*z*: M^+^ calcd for C_16_H_16_N_2_O_5_, 316.3086; found, 316.1051.

**(*S*)-*N*-(2,4-Bis((*E*)-3,3,3-trifluoroprop-1-en-1-yl)phenyl)-1-(2-(1,3-dioxo-2*H*-isoindolin-2-yl)acetyl)pyrrolidine-2-carboxamide (7)**: **5** (2 mmol) and lithium hydroxide (12 mmol) were placed in a flask. To the flask was added THF (4.8 mL), methanol (1.5 mL), water (1.5 mL), and the mixture was stirred overnight at room temperature. The resulting solution was concentrated in vacuo, the residue was diluted with aq HCl and, extracted with CHCl_3_:EtOH (2:1). The organic layer was washed with brine and dried over MgSO_4_. The solvent was removed in vacuo to give **6**. To a solution of **6** (1 mmol) in anhydrous CH_2_Cl_2_ was added oxalyl chloride (2 mmol) and anhydrous DMF (1 drop) at 0 °C, and mixture was stirred at room temperature. After the reaction mixture was stirred for 3 h, it was concentrated in vacuo. To the residue was added **1** (0.3 mmol), anhydrous 1,4-dioxane (3 mL) and, anhydrous pyridine (0.45 mmol), and mixture was stirred at room temperature. After the reaction mixture was stirred for 2 h, it was quenched with sat*.* NaHCO_3_ and, extracted with CHCl_3_. The CHCl_3_ layer was washed with water and dried over MgSO_4_. The solvent was removed in vacuo and the residue was purified by column chromatography to give (*S*)-*N*-(2,4-bis((*E*)-3,3,3-trifluoroprop-1-en-1-yl)phenyl)-1-(2-(1,3-dioxo-2*H*-isoindolin-2-yl)acetyl)pyrrolidine-2-carboxamide (**7**) in 31% yield (71.9 mg, 0.12 mmol). A white solid: mp 181–182 °C; ^1^H NMR (CDCl_3_) δ 1.87–1.97 (m, 1H), 2.14–2.28 (m, 2H), 2.63–2.67 (m, 2H), 3.58–3.77 (m, 2H), 4.51 (d, *J* = 3.9 Hz, 2H), 4.82 (d, *J* = 7.8 Hz, 1H), 6.11 (qd, *J* = 6.4, 16.0 Hz, 2H), 7.08 (qd, *J* = 2.2, 16.0 Hz, 1H), 7.28 (qd, *J* = 2.1, 16.0 Hz, 1H), 7.44 (d, *J* = 6.6 Hz, 2H), 7.73–7.90 (m, 4H), 8.08 (d, *J* = 9.0 Hz, 1H), 9.46 (s, 1H); ^13^C NMR (CDCl_3_) δ 26.3, 29.7, 39.7, 46.9, 61.2, 115.8 (q, *J* = 33.9 Hz), 119.4 (q, *J =* 34.0 Hz), 122.7 (q, *J* = 270.0 Hz), 122.8, 123.5 (q, *J* = 269.0 Hz), 123.8, 125.8, 126.4, 129.9, 132.0, 132.7 (q, *J* = 6.7 Hz), 136.4, 137.6 (q, *J* = 6.8 Hz), 137.6, 167.2, 167.8, 168.5; ^19^F NMR (CDCl_3_) δ 89.32 (dd, *J* = 2.2, 6.6 Hz, 3F), 89.54 (dd, *J* = 2.2, 6.4 Hz, 3F); MS *m*/*z*: M^+^ 565, 468, 285, 257, 188, 160; HRMS *m*/*z*: M^+^ calcd for C_27_H_21_F_6_N_3_O_4_, 565.1436; found, 595.1437.

***N*-(*S*)-1-(2-Aminoacetyl)-*N*-(2,4-bis((*E*)-3,3,3-trifluoroprop-1-en-1-yl)phenyl)pyrrolidine-2-carboxamide (H-Gly-Pro-1)**: To solution of **7** (0.05 mmol) in ethanol was added hydrazine hydrate (0.05 mmol), and mixture was stirred for 3 h at room temperature. The resulting precipitate was filtered, then the filtrate was concentrated in vacuo and the residue was purified by column chromatography to give *N*-(*S*)-1-(2-aminoacetyl)-*N*-(2,4-bis((*E*)-3,3,3-trifluoroprop-1-en-1-yl)phenyl)py-rolidine-2-carboxamide (H-Gly-Pro-**1**) in 72% yield (15.7 mg, 0.036 mmol). A light yellow solid: mp 129–130 °C; ^1^H NMR (DMSO-*d*_6_) δ 1.90–2.25 (m, 4H), 3.51–3.80 (m, 2H), 4.56 (d, *J* = 8.3 Hz), 6.75–6.95 (m, 2H), 7.21–7.53 (m, 4H), 7.75 (d, *J* = 8.2 Hz), 8.20 (s, 1H); ^13^C NMR (DMSO-*d*_6_) δ 24.8, 26.7, 46.2, 60.6, 70.3, 116.2 (q, *J* = 33.6 Hz), 117.2 (q, *J* = 33.3 Hz), 124.3 (q, *J* = 269.0 Hz), 124.6 (q, *J* = 269.0 Hz), 126.6, 127.2, 128.7, 130.4, 131.2, 134.1 (q, *J* = 7.0 Hz), 137.6 (q, *J* = 7.0 Hz), 138.4, 171.4; ^19^F NMR (DMSO-*d*_6_) δ 80.80 (dd, *J* = 2.2, 7.2 Hz, 3F), 80.91 (dd, *J* = 2.2, 7.2 Hz, 3F); MS *m*/*z*: M^+^ 435, 281, 242, 174, 155, 127; HRMS *m*/*z*: M^+^ calcd for C_19_H_19_F_6_N_3_O_2_, 435.3635; found, 435,1382.

### Assay of dipeptidyl peptidase activity using H-Gly-Pro-1 as a substrate

Stenotrophomonas dipeptidyl aminopeptidase IV was prepared from the E. coli DH5 containing pUC19-SDP4 as described previously [22]. The enzyme activity was assayed using H-Gly-Pro-**1** as a substrate following the procedure described previously [23]. The reaction mixture (total 100 μL) contained 0.1 M Tris–HCl (pH 8.0), 10–50 μM substrate and 10–400 ng of the enzyme. The reaction was initiated by the addition of the enzyme solution. Following incubation at 37 °C for 5 min in a 96-well plate inside a SH-9000 plate reader (Corona electric, Japan), the amount of **1** liberated was determined fluorometrically. The excitation and emission wavelengths used were 300 nm and 460 nm for **1**, respectively. The reaction velocities (µmol/min/mg protein) were compared.
